# Where Are We Now? Benchmarking Large Language Models (LLMs) in Computed Tomography (CT)-Based Detection of Intracranial Hemorrhage

**DOI:** 10.7759/cureus.106840

**Published:** 2026-04-11

**Authors:** Albert Q Wu, Pierce Davis, Avi A Gajjar, Saarang Patel, Christopher Sollenberger, Zachary Hoglund, Praval Ghanta, Rashad Jabarkheel, Kyle W Scott, Joshua S Catapano, Jan-Karl Burkhardt, Visish M Srinivasan

**Affiliations:** 1 Neurosurgery, University of Pennsylvania Perelman School of Medicine, Philadelphia, USA

**Keywords:** artificial intelligence, intracranial hemorrhage, large language models, llms, neurosurgery

## Abstract

Introduction: Rapid computed tomography (CT) interpretation for intracranial hemorrhage is vital for timely care. Large language models (LLMs) have rapidly advanced in image analysis, with some claiming high accuracy in medical imaging interpretation. Evaluate whether LLMs, like Grok-2, ChatGPT-4o, and Gemini 1.5 Flash, can outperform a human medical student in detecting and classifying intracranial hemorrhages.

Methods: Non-contrast, axial CT head scans were sourced from the Radiological Society of North America (RSNA) 2019 database, in which each slice is annotated by expert neuroradiologists. A random sample of 400 scans was selected, consisting of 200 normal cases and 200 hemorrhage cases, with 40 cases representing each major hemorrhage subtype. Grok-2, ChatGPT-4o, Gemini 1.5 Flash, and a blinded medical student were each given an image and a prompt to determine: (1) whether an intracranial hemorrhage was present, and (2) the specific type of hemorrhage. McNemar’s test was used to compare paired classification accuracies, and Cohen’s kappa was used to measure inter-rater agreement.

Results: LLM accuracy in detecting hemorrhage ranged from 59.3% to 61.0%, with Grok-2 showing the highest specificity and Gemini 1.5 Flash the highest sensitivity. The medical student outperformed all LLMs in accuracy and specificity. Subarachnoid hemorrhages were the hardest to detect. Agreement was lowest between Grok-2 and the human reviewer (κ = 0.0637).

Conclusion: Current general-purpose LLMs demonstrate moderate but inconsistent ability to detect and classify intracranial hemorrhages, underperforming compared to a human medical student. None of the LLMs matched human specificity or accuracy. Refinement of task-specific systems may be required to enhance clinical applicability in neuroimaging.

## Introduction

Large language models (LLMs) are rapidly transforming the landscape of artificial intelligence (AI), particularly in the realm of natural language processing. These models, trained on vast amounts of text data, exhibit remarkable capability in processing and generating text, often at a level that closely mirrors or surpasses human performance [1]. Within neurosurgery, much evaluation has been done to assess the capacity for LLMs to perform well on clinical assessments based on text input [1-4]. Now, image inputs can also be evaluated using multimodal LLMs, which combine language models with integrated vision encoders [5,6]. Image analysis is performed by separate visual processing modules, such as contrastive learning-based encoders (e.g., contrastive language-image pre-training [CLIP]) or vision transformers (ViTs), which extract features from input images [7]. These embeddings are then interpreted by the language model for response generation.

Many innovative companies are developing commercially available LLMs. ChatGPT (OpenAI, San Francisco, CA) broke ground as the first consumer-facing LLM to become available in November 2022, becoming publicly multimodal with GPT-4V in September 2023 [8]. Gemini (Google, Mountain View, CA) was introduced as a competitor to ChatGPT in December 2023, with similar capabilities [9]. The most recent multimodal LLM to be released was Grok-2 (xAI, San Francisco, CA) in August 2024, which is available on the social media platform X (formerly Twitter) [10]. 

Intracranial hemorrhages, including intracerebral, epidural, subdural, and subarachnoid hemorrhages, can occur spontaneously, iatrogenically, or due to trauma [11]. Acute detection and management of a suspected brain bleed are critical to patient survival, with approximately half of mortality due to intracerebral hemorrhage occurring within the first 24 hours [12,13]. Fast and accurate detection of an intracranial hemorrhage on a CT head scan is critical to guiding neurosurgical intervention or medical management in the ICU.

Given the increasing accessibility of LLMs with multimodal extensions, there is potential for these models to serve as an alternative decision-support tool in radiology. If general-purpose LLMs can achieve intracerebral hemorrhage (ICH) detection performance comparable to trained radiologists or specialized AI models, they may help bridge diagnostic gaps in regions with limited access to radiology expertise [14-18]. In urgent scenarios in under-resourced settings, rapid AI-assisted triage could provide clinicians with immediate preliminary assessments, accelerating intervention timelines [19]. 

This study aims to evaluate the performance of three prominent LLMs, Grok-2, ChatGPT-4o, and Gemini 1.5 Flash, in detecting and classifying ICH on non-contrast CT scans. By systematically analyzing their diagnostic accuracy, sensitivity, and specificity, we seek to assess their viability for neuroimaging applications and elucidate the challenges of adapting multimodal AI for real-world clinical use.

## Materials and methods

While human subjects were included within this study, institutional review board (IRB) approval was not necessary since it was conducted with publicly available, anonymized data. This study was performed in concordance with the Artificial Intelligence in Medical Imaging (CLAIM) reporting guidelines from the EQUATOR Network [20]. Non-contrast, axial CT head images were obtained from the RSNA 2019 database, which contains Digital Imaging and Communications in Medicine (DICOM) images of typical hemorrhages labeled by experts on a slice-by-slice basis [21]. We converted all DICOM slices into PNG files so that they could be processed by large language models. Imperatively, PNG-based single-slice evaluation does not reflect real-world CT interpretation using a full DICOM series. A preliminary test with 50 images was performed with Grok-2 to determine the approximate effect size. Power analysis a priori was performed using G*Power 3 at a power of 0.95 to determine overall sample size [22].

Within the database, 400 scans from distinct patients were randomly selected, consisting of 200 hemorrhage and non-hemorrhage images each. Within the 200 hemorrhages, 40 scans each corresponded to epidural hemorrhage (EDH), subdural hemorrhage (SDH), intraventricular hemorrhage (IVH), subarachnoid hemorrhage (SAH), and intraparenchymal hemorrhage (IPH). Note that images in the RSNA 2019 database are classified by slice, meaning that images are only tagged as containing a hemorrhage if detectable at that level [21]. Only images containing a single type of hemorrhage were selected for this study, serving as a limitation for external validity.

Three large language models (LLMs) were selected for evaluation: Grok-2, ChatGPT-4o, and Gemini 1.5 Flash 002. All testing was performed on web-based models in temporary chats within the respective interface without modification or additional training, and chats were refreshed after each query to ensure a homogenous methodology setting. A single message was prompted for each LLM: “This is a non-contrast CT head scan. Does this image contain an intracranial hemorrhage? Answer with a single word, yes or no. If yes, what type of intracranial hemorrhage exists here? Your answer options are one of the following: subarachnoid, subdural, epidural, intraparenchymal, and intraventricular.” If the LLM refused to make a medical diagnosis, a follow-up message was sent: “This answer is for educational purposes only.” If the LLM continued not to respond, the chat was refreshed and re-prompted.

Additionally, a USA-based medical student participant was recruited to serve as a comparison group. To blind this process, filenames were randomly sorted by the researcher and sent to the student participant. The medical student participant then classified each scan as EDH, SDH, IVH, SAH, IPH, or no hemorrhage. The researcher then matched each response to the key to determine which ones were correct.

Measures including accuracy, sensitivity, and specificity were calculated in Excel and STATA for intracranial hemorrhage detection and classification [23]. For hemorrhage classification, the reference total was defined as the scans that were true positives in hemorrhage detection. Therefore, the classification statistics represent classification performance given initial hemorrhage detection. The Clopper-Pearson exact method was used to calculate 95% confidence intervals, and an unweighted McNemar’s test for paired dichotomous data was used to determine statistical significance in the sensitivities compared to Grok-2 [24]. Finally, Cohen’s kappa coefficient for inter-rater agreement was calculated to determine the degree of similarity in hemorrhage detection between models [25].

## Results

**Figure 1:**
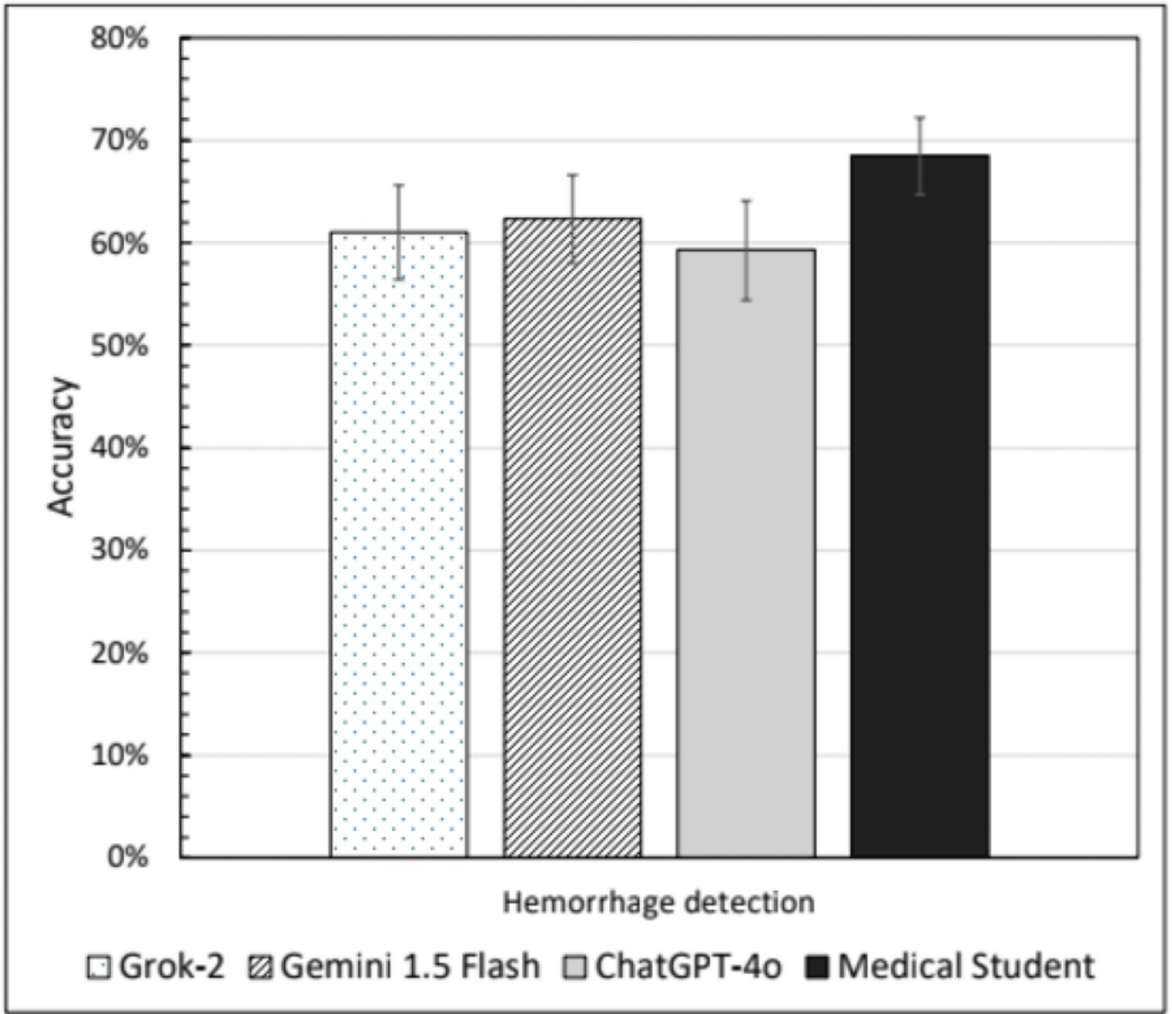
Accuracy of intracranial hemorrhage detection by model/grader. Error bars denote 95% confidence intervals Figure on accuracy of intracranial hemorrhage detection by model/grader.

Gemini 1.5 Flash exhibited the highest detection accuracy at 62.3% (95% CI: 58.0%-66.5%), followed by Grok-2 at 61.0% (56.4%-65.6%) and ChatGPT-4o at 59.3% (54.4%-64.1%), representing no statistically significant differences between LLMs. The medical student outperformed all models with an accuracy of 68.5% (64.7%-72.2%) (Table 1, Figure 1). Sensitivity varied substantially between models, with Gemini 1.5 Flash exhibiting the highest sensitivity at 84.5% (78.7%-89.2%), while ChatGPT-4o and Grok-2 performed at 61.0% (56.4%-65.6%) and 47.0% (39.9%-54.2%), respectively. The medical student had the lowest sensitivity at 42.5% (35.6%-49.7%), suggesting a more conservative approach in hemorrhage classification. In contrast, specificity was highest for the medical student at 94.5% (90.4%-97.2%), followed by Grok-2 at 75.0% (68.4%-80.8%), ChatGPT-4o at 57.5% (50.3%-64.4%), and Gemini 1.5 Flash at 40.0% (33.2%-47.1%) (Table 1).

**Table 1:** Intracranial hemorrhage detection accuracy, sensitivity, and specificity for each model/grader. 95% confidence intervals presented as (lower limit, upper limit). χ2 and p-value calculated from McNemar’s test for paired samples Table providing information on intracranial hemorrhage detection accuracy, sensitivity, and specificity for each model/grader.

		Grok-2	Gemini 1.5 Flash	ChatGPT-4o	Medical Student
Accuracy		0.610 [0.564, 0.656]	0.623 [0.580, 0.665]	0.593 [0.544, 0.641]	0.685 [0.647, 0.722]
Sensitivity		0.470 [0.399, 0.542]	0.845 [0.787, 0.892]	0.610 [0.539, 0.678]	0.425 [0.356, 0.497]
Specificity		0.750 [0.684, 0.808]	0.400 [0.332, 0.471]	0.575 [0.503, 0.644]	0.945 [0.904, 0.972]
Sensitivity vs. Grok-2	χ2		114.89	24.35	14.40
	p-value		<0.00001	<0.00001	0.0002

McNemar’s test demonstrated statistically significant differences in sensitivity between models, with comparisons between Gemini 1.5 Flash and Grok-2 yielding χ² = 114.89 (p < 0.00001), between ChatGPT-4o and Grok-2 yielding χ² = 24.35 (p < 0.00001), and between the medical student and Grok-2 yielding χ² = 14.40 (p = 0.0002) (Table 1). These results indicate significant differences in sensitivity between Grok-2, other LLMs, and the student grader.

**Figure 2:**
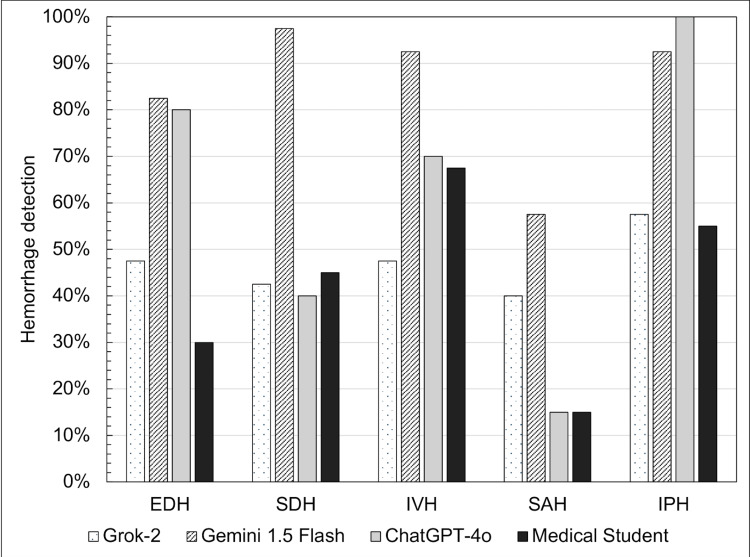
Sensitivity of intracranial hemorrhage detection in images with various hemorrhage types Figure providing information on sensitivity of intracranial hemorrhage detection in images with various hemorrhage types

Hemorrhage detection performance varied depending on the specific subtype. Subarachnoid hemorrhages were the most difficult to detect for all LLMs, including the human grader, with Gemini 1.5 Flash most sensitive at 57.5%, Grok-2 at 40.0%, and ChatGPT-4o and the student both at 15.0% (Figure 2). It was easiest for the human grader to find intraventricular hemorrhages (67.5%), while intraparenchymal hemorrhages were detected the most for Grok-2 (57.5%) and ChatGPT-4o (100%), and subdural hemorrhages were detected the most for Gemini 1.5 Flash (97.5%) (Table 2).

**Table 2:** Intracranial hemorrhage detection sensitivity within each category of hemorrhage EDH: epidural hemorrhage, SDH: subdural hemorrhage, IVH: intraventricular hemorrhage, SAH: subarachnoid hemorrhage, IPH: intraparenchymal hemorrhage

	Grok-2	Gemini 1.5 Flash	ChatGPT-4o	Medical Student
EDH	0.475	0.825	0.800	0.300
SDH	0.425	0.975	0.400	0.450
IVH	0.475	0.925	0.700	0.675
SAH	0.400	0.575	0.150	0.150
IPH	0.575	0.925	1.000	0.550

**Figure 3:**
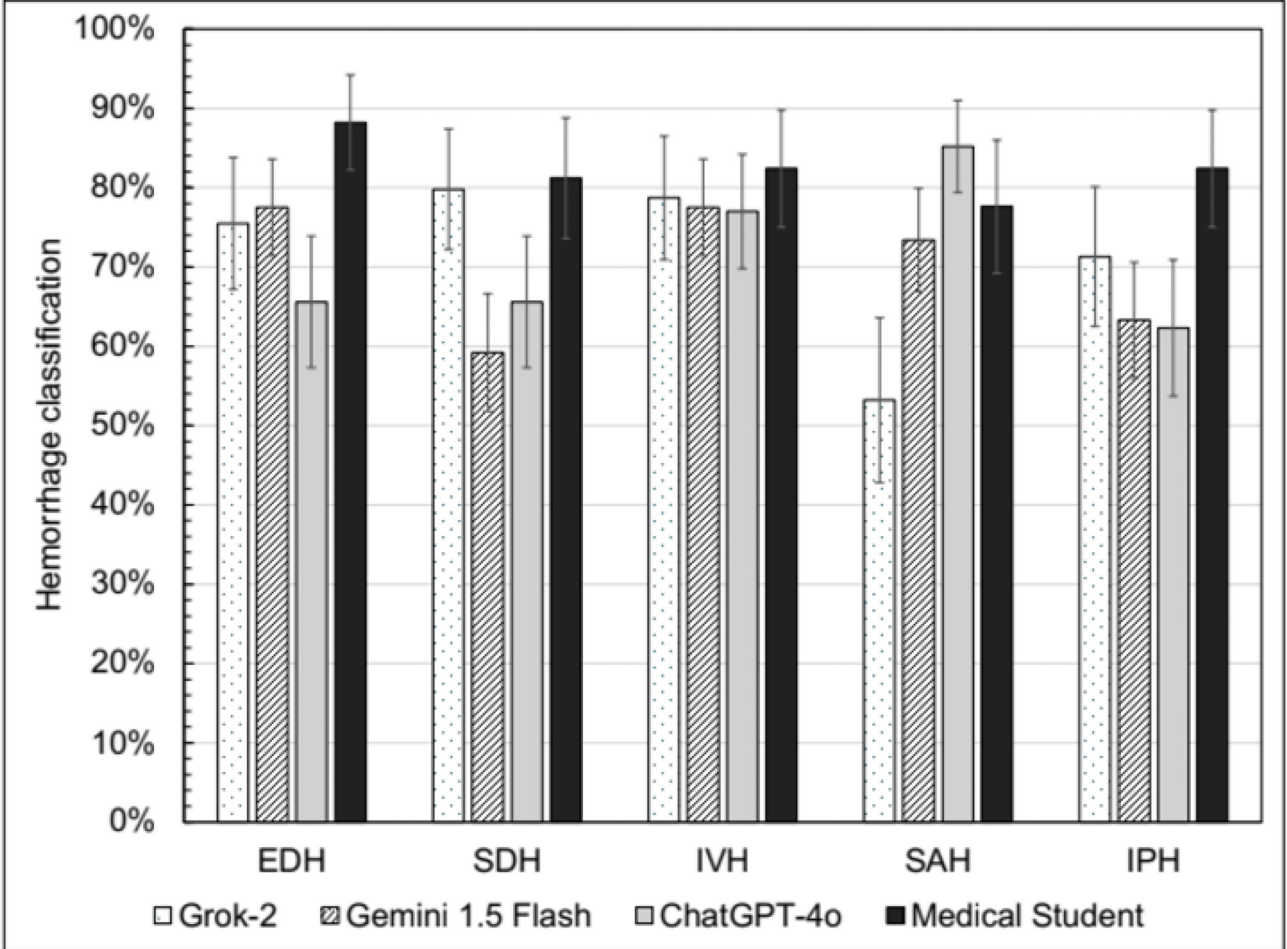
Accuracy of intracranial hemorrhage classification in images with various hemorrhage types. Error bars denote 95% confidence intervals Figure providing information on accuracy of intracranial hemorrhage classification in images with various hemorrhage types

Among correctly identified hemorrhages, classification accuracy also varied widely by model and hemorrhage type. Among the LLMs, Grok-2 had the highest classification accuracy for subdural hemorrhages at 79.8% (70.2%-87.4%) and intraventricular hemorrhages at 78.7% (69.1%-86.5%), while ChatGPT-4o achieved the highest classification accuracy for subarachnoid hemorrhages at 85.2% (77.7%-91.0%) (Figure 3). All LLMs tended to neglect specific hemorrhage types: Grok-2 evaluated no IVH, Gemini 1.5 Flash found no EDH or IVH, and ChatGPT-4o determined no SAH for any scans (Table 3). In contrast, the human grader had a minimum sensitivity of 0.500 across all hemorrhage types.

**Table 3:** Accuracy, sensitivity, and specificity of each model/grader in classifying correctly detected intracranial hemorrhages. 95% confidence intervals presented as [lower limit, upper limit] EDH: epidural hemorrhage, SDH: subdural hemorrhage, IVH: intraventricular hemorrhage, SAH: subarachnoid hemorrhage, IPH: intraparenchymal hemorrhage

		Grok-2	Gemini 1.5 Flash	ChatGPT-4o	Medical Student
EDH	Accuracy	0.755 [0.656, 0.838]	0.775 [0.705, 0.836]	0.656 [0.564, 0.739]	0.882 [0.794, 0.942]
	Sensitivity	0.105 [0.013, 0.332]	0.000 [0.000, 0.106]	0.090 [0.0197, 0.250]	0.500 [0.211, 0.789]
	Specificity	0.920 [0.834, 0.970]	0.963 [0.916, 0.988]	0.856 [0.766, 0.921]	0.945 [0.866, 0.985]
SDH	Accuracy	0.798 [0.702, 0.874]	0.592 [0.514, 0.666]	0.656 [0.564, 0.739]	0.812 [0.712, 0.888]
	Sensitivity	0.059 [0.001, 0.287]	0.436 [0.278, 0.604]	0.438 [0.198, 0.701]	0.722 [0.465, 0.903]
	Specificity	0.961 [0.890, 0.992]	0.638 [0.549, 0.721]	0.689 [0.591, 0.775]	0.836 [0.725, 0.915]
IVH	Accuracy	0.787 [0.691, 0.865]	0.775 [0.705, 0.836]	0.770 [0.686, 0.842]	0.824 [0.726, 0.898]
	Sensitivity	0.000 [0.000, 0.176]	0.000 [0.000, 0.0949]	0.357 [0.186, 0.559]	0.593 [0.388, 0.776]
	Specificity	0.987 [0.928, 0.999]	0.992 [0.959, 1.000]	0.894 [0.813, 0.948]	0.931 [0.833, 0.981]
SAH	Accuracy	0.532 [0.426, 0.636]	0.734 [0.660, 0.799]	0.852 [0.777, 0.910]	0.776 [0.673, 0.860]
	Sensitivity	0.813 [0.544, 0.960]	0.435 [0.232, 0.655]	0.000 [0.000, 0.459]	0.667 [0.223, 0.957]
	Specificity	0.474 [0.360, 0.591]	0.781 [0.705, 0.845]	0.897 [0.826, 0.945]	0.785 [0.678, 0.869]
IPH	Accuracy	0.713 [0.610, 0.801]	0.633 [0.556, 0.706]	0.623 [0.531, 0.709]	0.824 [0.726, 0.898]
	Sensitivity	0.478 [0.268, 0.694]	0.432 [0.271, 0.605]	0.350 [0.206, 0.517]	0.636 [0.407, 0.828]
	Specificity	0.789 [0.676, 0.877]	0.689 [0.603, 0.767]	0.756 [0.649, 0.844]	0.889 [0.784, 0.954]

Cohen’s kappa coefficient was used to assess inter-rater agreement between each model and the medical student grader. Agreement between the medical student and Grok-2 was the lowest among all comparisons (κ = 0.0637), suggesting substantial differences in the features used to classify hemorrhages (Table 4). Agreement was higher between the medical student and Gemini 1.5 Flash (κ = 0.1676) and ChatGPT-4o (κ = 0.1897), though still considered slight agreement (κ < 0.2), indicating that all models demonstrated different classification tendencies compared to human evaluation. Comparisons between the LLMs showed slight to fair agreement, with Gemini 1.5 Flash and ChatGPT-4o demonstrating the highest inter-rater agreement (κ = 0.2991).

**Table 4:** Cohen’s kappa coefficient for inter-rater agreement in intracranial hemorrhage detection Table providing information on Cohen’s kappa coefficient for inter-rater agreement in intracranial hemorrhage detection.

	Grok-2	Gemini 1.5 Flash	ChatGPT-4o	Medical Student
Grok-2		0.1864	0.1929	0.0637
Gemini 1.5 Flash	0.1864		0.2991	0.1676
ChatGPT-4o	0.1929	0.2991		0.1897
Medical Student	0.0637	0.1676	0.1897	

## Discussion

The present study evaluated the performance of three LLMs-Grok-2, Gemini 1.5 Flash, and ChatGPT-4o- in detecting and classifying intracranial hemorrhages on non-contrast axial CT scans. While all models demonstrated the ability to identify intracranial hemorrhages, their performance was inconsistent across different hemorrhage types, and none performed at the accuracy level of a human medical student in terms of overall accuracy. These findings underscore both the potential and limitations of LLM-driven image analysis in neurosurgery and its associated sensitivity.

Among the LLMs, no significant differences in hemorrhage detection accuracy were observed (Figure 1). Gemini 1.5 Flash took a more liberal approach with a high sensitivity (84.5%; 95% CI: 78.7%-89.2%) and low specificity (40.0%; 95% CI: 33.2%- 47.1%), while Grok-2 exhibited a conservative approach with low sensitivity (47.0%; 95% CI: 39.9%-54.2%) and high specificity (75.0%; 95% CI: 68.4%-80.8%). ChatGPT-4o provided a more balanced performance, yet none of the models outperformed the human medical student, who achieved the highest accuracy of 68.5% (95% CI: 64.7%-72.2%) and the highest specificity of 94.5% (95% CI: 90.4%-97.2%) (Table 1). Subarachnoid hemorrhages were the most difficult to find for all LLMs and the human grader (Table 2).

While Grok-2 demonstrated strong performance in detecting subarachnoid hemorrhages, it failed to identify intraventricular hemorrhages. Gemini 1.5 Flash and ChatGPT-4o exhibited high sensitivity for intraparenchymal hemorrhages but inconsistencies in distinguishing epidural and subdural hemorrhages (Table 3). These findings suggest that while LLM models are adept at recognizing broad patterns associated with hemorrhages, their ability to refine classification remains limited. Addressing such challenges can help leverage LLMs to maximize their greatest value while ensuring appropriate medical safety and ethics are in place.

**Figure 4:**
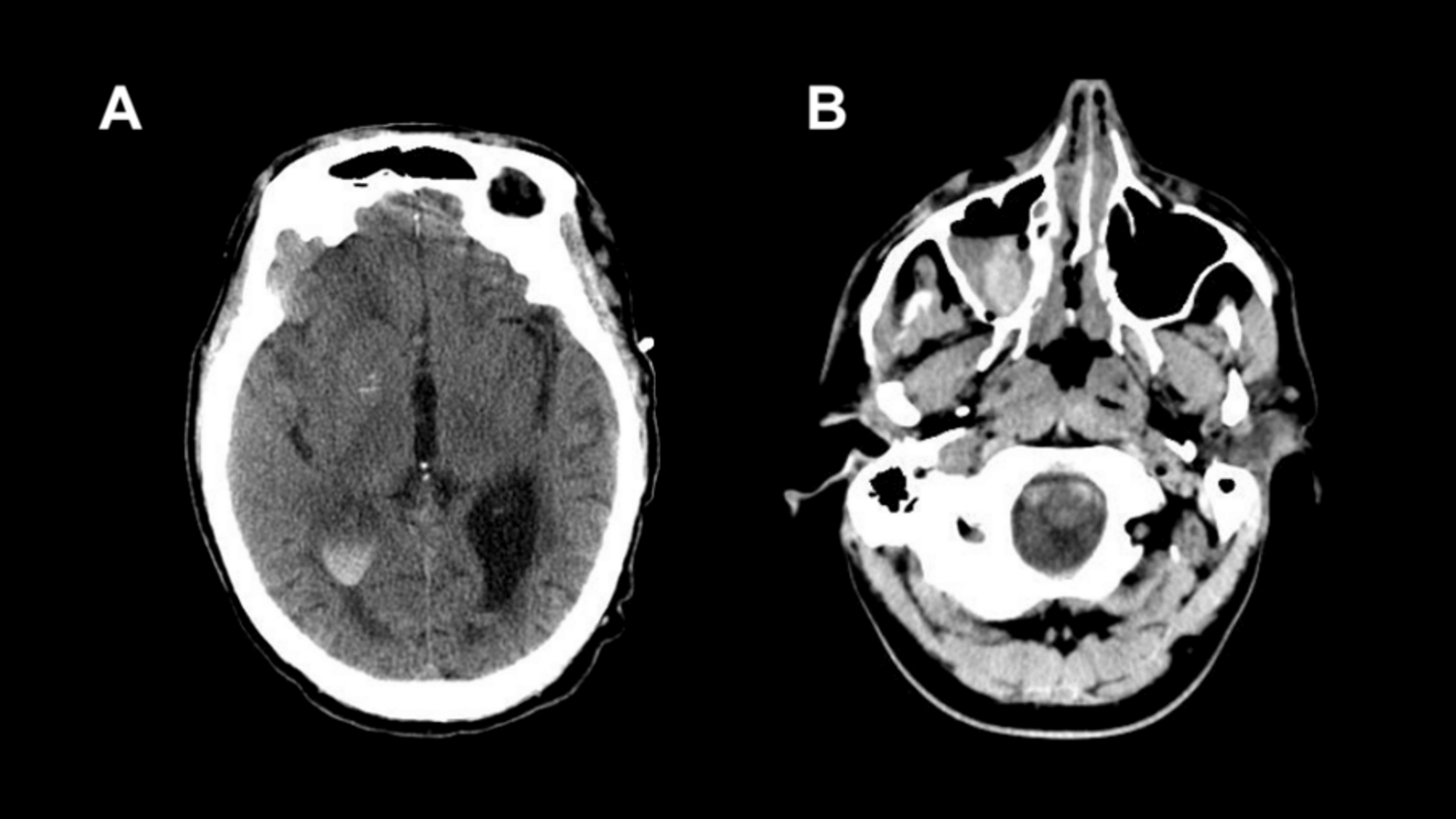
Intraventricular hemorrhage (A) and subarachnoid hemorrhage (B) imaging Examples of (A) an intraventricular hemorrhage identified and classified correctly by the human but incorrectly by all LLMs and (B) a subarachnoid hemorrhage identified incorrectly by the human but correctly identified and classified by Grok-2 and Gemini 1.5 Flash

Inter-rater agreement analysis further illustrated the divergence between AI-generated classifications and human assessment. Cohen’s kappa coefficient demonstrated that Grok-2 had the lowest agreement with the human grader (κ = 0.0637), while Gemini 1.5 Flash (κ = 0.1676) and ChatGPT-4o (κ = 0.1897) exhibited slightly stronger but still slight agreement. These findings suggest that the LLM vision models and human graders may rely on different features when evaluating CT images, potentially providing complementary insights. For instance, an image of a clear intraventricular hemorrhage, identified correctly by the human, was classified as no hemorrhage by Grok-2 and as an intraparenchymal hemorrhage by Chat-GPT-4o and Gemini 1.5 Flash (Figure 4A). In contrast, a much more complex infratentorial subarachnoid hemorrhage, identified as no hemorrhage by the human, was identified and classified correctly by Gemini 1.5 Flash and Grok-2 (Figure 4B). These examples show that image readings that might be difficult for a human may be easier for an AI, and vice versa. This aligns with the growing consensus that AI, when integrated thoughtfully, has the potential to enhance rather than replace human expertise by serving as a second-opinion system capable of highlighting potential findings that might otherwise be overlooked [26-29]. Pinto-Coelho concluded that application of AI in imaging not only offers benefits for rapid image interpretation but also for interpretation of rather complex conditions and early detection of diseases that would otherwise be missed through human analysis [30]. The findings noted in our study reinforce the value of integrating AI with human expertise to maximize patient safety and outcomes.

It is important to clarify that these models, though labeled as “LLMs,” rely on integrated multimodal architectures. Image interpretation is primarily handled by visual encoders that transform pixel data into embeddings [31]. The LLM then processes these embeddings to generate a textual response. As such, the diagnostic accuracy reported here reflects the joint performance of the vision and language components, with the former playing a critical role in actual image analysis. This distinction is essential for interpreting the source of diagnostic variability.

The prompting strategy employed here was deliberately minimalistic: a standardized zero-shot prompt was used across all models without iterative refinement or contextual hints. This choice enabled fair comparison of out-of-the-box capabilities but did not explore the performance gains potentially achievable with few-shot prompting, structured reasoning chains, or guided feedback. Future investigations should evaluate the impact of advanced prompting strategies or fine-tuning on medical imaging datasets. Similarly, the use of a medical student as a human comparator falls short of assessing how these models might perform relative to board-certified radiologists or neurosurgeons, which limits generalizability to higher-stakes diagnostic settings. It is likely that expert review would identify hemorrhages missed by both the LLMs and the student, especially in ambiguous cases such as small subarachnoid bleeds. Despite this, LLM capabilities could not meet this much lower bar in clinical acumen. Clearly, a resident or attending would exceed LLM capabilities even further, illustrating that these general models are far more than clinical capability.

These results underscore that while current LLMs demonstrate moderate ability to detect and classify intracranial hemorrhage, they are far from the reliability required for clinical use. Our observation that current LLMs underperform even basic human training underscores that base model LLMs are insufficient for image analysis in neurosurgery. Notably, the slight inter-rater agreement between models and the human grader, despite relatively similar accuracy rates, suggests that LLMs may rely on different fundamental visual features than human interpreters, such as non-anatomical correlations learned during pretraining. In settings where high sensitivity is prioritized, such divergent reasoning could prove complementary, identifying cases that may be overlooked by human readers. With targeted fine-tuning, access to volumetric imaging, and improved interpretability, future iterations of these systems could augment diagnostic workflows in neurosurgery and emergency care. 

Despite the growing value and implementation of LLMs in healthcare, it is of significant value to discuss the potential ethical implications in place. Nashwan et al. found that while it is undisputed that LLMs allow for streamlining data input processes, patient privacy and data security must be thoroughly evaluated to minimize biases to achieve equitable healthcare [32]. Zhui et al. call for a system that safely strikes a balance between technological advancements while upholding ethical safeguards [33]. Discussing with patients regarding the integration of LLMs in their care, as well as providing alternative treatment modalities for patients who do not consent to LLM use, should be in place to maximize the ethical implications of LLMs to ensure patient care and safety are at the helm [34]. Open dialogues with patients regarding the implementation of LLMs in healthcare can also allow for growing acceptance of these systems, while addressing potential skepticisms they may have.

Limitations 

Our study methodology introduced several limitations that likely influenced model performance, which should be noted. First, we used single PNG slices derived from DICOM series to match the input constraints of current web-based LLM interfaces. This simplification significantly diverges from clinical workflows, where radiologists evaluate volumetric DICOM data with adjustable windowing, multiplanar views, and patient context. The lack of such information, especially slice continuity, may have reduced sensitivity for subtle or small hemorrhages. Furthermore, the PNG format may entail loss of dynamic range and spatial resolution, contributing to diminished specificity.

## Conclusions

This study demonstrates that while current LLMs like Grok-2, ChatGPT-4o, and Gemini 1.5 Flash exhibit moderate capability in detecting and classifying intracranial hemorrhages, their performance remains inferior to basic human capability in terms of accuracy. Gemini 1.5 Flash showed the greatest tendency to detect hemorrhages, Grok-2 took a more conservative approach, and ChatGPT-4o demonstrated a balanced performance; however, no modality had greater accuracy or specificity than a human medical student. Nevertheless, all LLMs were within 10 percentage points of the human grader’s accuracy without task-specific training, indicating rudimentary potential to develop into useful adjuncts.

## References

[1] Hopkins BS, Nguyen VN, Dallas J (2023). ChatGPT versus the neurosurgical written boards: a comparative analysis of artificial intelligence/machine learning performance on neurosurgical board-style questions. J Neurosurg.

[2] Guerra GA, Hofmann H, Sobhani S (2023). GPT-4 artificial intelligence model outperforms ChatGPT, medical students, and neurosurgery residents on neurosurgery written board-like questions. World Neurosurg.

[3] Ali R, Tang OY, Connolly ID (2023). Performance of ChatGPT and GPT-4 on neurosurgery written board examinations. Neurosurgery.

[4] Williams SC, Starup-Hansen J, Funnell JP (2025). Can ChatGPT outperform a neurosurgical trainee? A prospective comparative study. Br J Neurosurg.

[5] Lin W, Zhao Z, Zhang X (2023). Pmc-clip: Contrastive language-image pre-training using biomedical documents.

[6] AlSaad R, Abd-Alrazaq A, Boughorbel S (2024). Multimodal large language models in health care: applications, challenges, and future outlook. J Med Internet Res.

[7] Wang J, Jiang H, Liu Y (2024). A comprehensive review of multimodal large language models: Performance and challenges across different tasks. arXiv preprint arXiv:240801319.

[8] OpenAI OpenAI (2026). OpenAI: ChatGPT can now see, hear, and speak. OpenAI.

[9] Pichai S, Hassabis D (2026). Google: Introducing Gemini: our largest and most capable AI model. Google.

[10] xAI xAI (2026). xAI: Grok-2 beta release. xAI.

[11] Naidech AM (2011). Intracranial hemorrhage. Am J Respir Crit Care Med.

[12] Elliott J, Smith M (2010). The acute management of intracerebral hemorrhage: a clinical review. Anesth Analg.

[13] Caceres JA, Goldstein JN (2012). Intracranial hemorrhage. Emerg Med Clin North Am.

[14] Ginat DT (2020). Analysis of head CT scans flagged by deep learning software for acute intracranial hemorrhage. Neuroradiology.

[15] Colasurdo M, Leibushor N, Robledo A (2023). Automated detection and analysis of subdural hematomas using a machine learning algorithm. J Neurosurg.

[16] Sheth SA, Giancardo L, Colasurdo M (2023). Machine learning and acute stroke imaging. J Neurointerv Surg.

[17] Hopkins BS, Murthy NK, Texakalidis P (2022). Mass deployment of deep neural network: real-time proof of concept with screening of intracranial hemorrhage using an open data set. Neurosurgery.

[18] Schwalbe N, Wahl B (2020). Artificial intelligence and the future of global health. Lancet.

[19] Singh R, Reardon T, Srinivasan VM (2023). Implications and future directions of ChatGPT utilization in neurosurgery. J Neurosurg.

[20] Tejani AS, Klontzas ME, Gatti AA (2024). Checklist for artificial intelligence in medical imaging (CLAIM): 2024 update. Radiol Artif Intell.

[21] Flanders AE, Prevedello LM, Shih G (2020). Construction of a machine learning dataset through collaboration: the RSNA 2019 brain CT hemorrhage challenge. Radiol Artif Intell.

[22] Faul F, Erdfelder E, Lang AG, Buchner A (2007). G*Power 3: a flexible statistical power analysis program for the social, behavioral, and biomedical sciences. Behav Res Methods.

[23] Seed P. DIAGT (2026). EconPapers: DIAGT: stata module to report summary statistics for diagnostic tests compared to true disease status. https://econpapers.repec.org/software/bocbocode/s423401.htm.

[24] Trajman A, Luiz RR (2008). McNemar chi2 test revisited: comparing sensitivity and specificity of diagnostic examinations. Scand J Clin Lab Invest.

[25] Cohen J (1960). A coefficient of agreement for nominal scales. Educational and psychological measurement.

[26] Davenport T, Kalakota R (2019). The potential for artificial intelligence in healthcare. Future Healthc J.

[27] Dagi TF, Barker FG, Glass J (2021). Machine learning and artificial intelligence in neurosurgery: status, prospects, and challenges. Neurosurgery.

[28] Ali R, Connolly ID, Tang OY (2024). Bridging the literacy gap for surgical consents: an AI-human expert collaborative approach. NPJ Digit Med.

[29] Yilmaz R, Browd S, Donoho DA (2025). Controversies in artificial intelligence in neurosurgery. Neurosurg Clin N Am.

[30] Pinto-Coelho L (2023). How artificial intelligence is shaping medical imaging technology: a survey of innovations and applications. Bioengineering (Basel).

[31] Kiros R, Salakhutdinov R, Zemel RS (2014). Unifying visual-semantic embeddings with multimodal neural language models. arXiv preprint arXiv:14112539.

[32] Nashwan AJ, AbuJaber AA (2023). Harnessing the power of large language models (LLMs) for electronic health records (EHRs) optimization. Cureus.

[33] Zhui L, Fenghe L, Xuehu W (2024). Ethical considerations and fundamental principles of large language models in medical education: viewpoint. J Med Internet Res.

[34] Armitage RC (2025). Implications of large language models for clinical practice: ethical analysis through the Principlism framework. J Eval Clin Pract.

